# Synchronous and multiple renal cell carcinoma, clear cell and papillary: An approach to clinically significant genetic abnormalities

**DOI:** 10.1590/S1677-5538.IBJU.2019.0015

**Published:** 2020-01-10

**Authors:** Laura Cifuentes-C, Carlos Humberto Martínez, Herney Andrés García-Perdomo

**Affiliations:** 1 Universidad Cooperativa de Colombia Pasto Colombia GIOD - Research Group Universidad Cooperativa de Colombia. Pasto, Colombia;; 2 Department of Surgery Division of Urology and Surgical Oncology Hospital Pablo Tobón Uribe MedellínAntioquia Colombia Department of Surgery, Division of Urology and Surgical Oncology, Hospital Pablo Tobón Uribe, Medellín, Antioquia, Colombia;; 3 School of Medicine Universidad del Valle Cali Colombia School of Medicine, UROGIV - Research Group, Universidad del Valle Cali, Colombia

## INTRODUCTION

Renal Cell Cancer (RCC) is a heterogeneous disease that is characterized by distinct pathological phenotypes due to the differences in genetic alterations and signaling pathways affected (1). Bilateral renal tumors are often thought to be familial, however, they are only found in 14% of RCC cases and 4% of von Hippel-Lindau disease (VHL) cases. Therefore, most people with bilateral kidney tumors might have sporadic tumors caused by somatic mutations (2). These figures suggest that a deep genomic study is fundamental for understanding the cause of this disease.

The objective of this report was to describe a clinical case of a patient with bilateral kidney tumors of different subtypes, and how the genetic abnormalities found in this patient relate to the clinical phenotype.

## CASE REPORT

**Figure 1:**
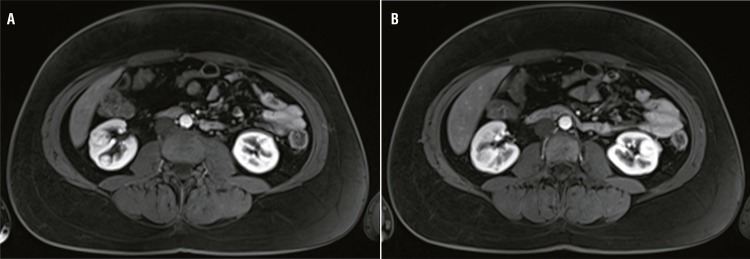
Abdominal Magnetic resonance. **a)** Right renal mass. b. Left renal mass.

We report the case of a 43 years old female patient with gross hematuria and renal colic who was evaluated by a community urologist. An abdominal non-contrast CT showed small kidney stones without obstruction and a left 14mm renal mass. She underwent abdominal magnetic resonance imaging (MRI), which revealed two right hyper-intense masses (9×7mm and 6×5mm) and one left mass (16×9mm), which was associated with one unspecific left pulmonary node (Figures 1a and 1b). These findings were evaluated at the uro-oncology clinic in March 2013. Initially, surgery was not recommended considering the young age of the patient and the small size of the tumors (less than 3cm). Furthermore, since this was a case of bilateral renal cell carcinoma (RCC), preserving renal function was important for future treatments. An active surveillance protocol with strict follow-up instructions was established according to the recommendations set by the National Cancer Institute. In September 2013, the MRI revealed that the left mass had grown to a volume of 21x15mm, but other tumors did not grow according to the imaging results.

A genetic test was performed in June 2013. This test specifically genotyped single nucleotide polymorphisms (SNPs) that were relevant to: (a) pharmaceutical drug response (b) genetic diseases and (c) complex diseases. In the latter group, colorectal, lung and breast cancer were included. Results from this genotyping test are presented in Table-1.

**Table 1:** SNP genotyping results associated with some cancer types.

	Gen/Locus	Marker	Genotype
Colorectal Cancer	BMP4	rs4444235	T/C
	CDH1	rs9929218	G/G
	CRAC1	rs4779584	T/C
	EIF3H	rs16892766	A/A
	Intergenic_10p14	rs10795668	G/G
	Intergenic_20p12	rs961253	C/C
	Intergenic_8q24 region3	rs6983267	G/G
	LOC120376	rs3802842	A/C
	RHPN2	rs10411210	C/C
	SMAD7	rs4939827	T/T
Lung Cancer	BAT3	rs3117582	A/A
	CHRNA3	rs1051730	T/C
	TERT	rs2736100	C/C
Breast Cancer	AKAP9	rs6964587	T/G
	CASP8	rs1045485	G/G
	CHEK2 1100delC		C/C
	ESR1	rs2046210	G/G
	FGFR2	rs1219648	A/A
	Intergenic_2q35	rs13387042	G/G
	Intergenic_8q24	rs13281615	A/G
	LSP1	rs3817198	T/T
	MAP3K1	rs889312	A/A
	MRPS30	rs10941679	A/G
	PALB2 1592delT		T/T
	TNRC9	rs3803662	C/C

In January 2014, we asked for a genetic sequence specific for renal cancer. We carried out a very extensive genomic sequencing study to identify polymorphisms associated with renal cancer and Birt-Hogg-Dubé (BHD) syndrome. This study sequenced a panel of five genes: the VHL gene, which has been associated with von Hippel-Lindau disease (3, 4), the MET gene, which is associated with papillary carcinoma of kidney cells (5, 6), genes SDHB and SDHD, which are associated with hereditary paraganglioma-pheochromocytoma (7, 6), and the FLCN gene, which is associated with BHD syndrome (8, 9). Predictive studies in silico of variants with unknown significance were performed using Alamut software V.2.11 (Interactive Biosoftware, http://www.interactive-biosoftware.com). Results from this sequencing are shown in Table-2.

**Table 2:** Sequence variants found in VHL, MET, FLCN, SDHB, and SDHD gene sequencing.

Gene	IDs.	HGVS	Patient	Functional consequence	Clinical Significance
SDHB	rs10887990	c.286+169A>G ^1^	C	Intron variant	Likely benign
SDHB	rs732679	c.73-302G>A ^1^	A	Intron variant	Likely benign
SDHB	rs2746462	c.18C>A ^1^	A	Synonymous codon	Benign
VHL	rs779806	c.340+384G>A ^2^	A	Intron variant	Likely benign
MET	rs34822187	c.1201-6898delA^3^	C	Intron variant	Likely benign
MET	rs41736, COSM150377	c.3912C>T ^3^ p.D1304D	T	Synonymous codon	Benign
MET	rs2023748, COSM150378	c.4071G>A^3^ p.A1357A	A	Synonymous codon	Benign
MET	rs41737, COSM150379	c.4146G>A ^3^ p.P1382P	A	Synonymous codon	Benign
FLCN	rs8065832	c.1062+6C>T ^4^	T	Intron variant	Unknown
FLCN	rs2018781	c.872-610C>G ^4^	G	Intron variant	Unknown
FLCN	rs1736219	c.397-14C>T ^4^	T	Intron variant	Benign
FLCN	rs1736212	c.-25+100C>G ^4^	G	Intron variant	Benign

Transcript of reference BIOBASE:(1) NM_003000.2, (2) NM_000551.3, (3) NM_001127500.1, (4) NM_144997.5

As shown in Table-2, a total of twelve variants were found in the patient. These variants were already reported in the literature. Out of these twelve, four are localized to the coding region of the analyzed genes. However, they are silent mutations since they do not change the sequence of the encoded protein. The remaining eight mutations were intron-located variants. Six of the eight were deep intronic variants, which are generally considered innocuous. The other two (rs8065832 y rs2018781) are located near splicing sites, which makes them clinically relevant but still unknown. These variants were analyzed using an in silico splicing analysis tool to predict whether they cause splicing defects (Table-3).

**Table 3:** In silico splicing analysis of the intronic variants of unknown clinical relevance.

Variant	SpliceSiteFinder [0-100]	MaxEntScan [0-12]	NNSplice [0-1]	GeneSplicer [0-15]	Human Splicing Finder [0-100]
rs8065832 (c.1062+6C>T)	SD: 84.14-89.92 (+6.9%)	SD: 9.88-10.28 (+4.1%)	SD: 0.88-0.90 (+2.1%)	SD: 9.40-11.20 **(+19.2%)**	SD: 47.34-74.17 **(+56.68%)**
rs2018781 (c.397-14C>T)	SA: 84.42–87.19 (+3.3%)	SA: 10.50-9.56 (-8.9%)	SA: 0.89-0.90 (+0.6%)	SA: 5.59-5.51 (-1.4%)	No difference between mutant and reference sequence was found.

Five different splice site prediction algorithms were used: (a) Splice Site Finder - SSF (http://www.interactive-biosoftware.com), (b) Gene Splicer -GS (http://www.tigr.org/tdb/GeneSplicer/gene_spl.html), (c) Splice site prediction by Neural Network - NNS (http://www.fruitfly.org/seq_tools/splice.html), (d) MaxEntScan–MES (http://genes.mit.edu/burgelab/maxent/Xmaxentscan_scoreseq.html), and (e) Human Splicing Finder - HSF (http://www.umd.be/HSF3/). SSF, MES, GS, and NNS were run simultaneously using the Alamut V.2.11. Default thresholds were used for all the analyses. The results of these in silico analyzes are shown in Table-3.

The in silico analyses (Table-3) predicted that the variant rs2018781 most likely has probably no impact on splicing. However, for the variant rs8065832, two of the algorithms predicted a higher score than the natural splicing site score (> 10%). According to the criteria by Thery et al., this result indicates that this variant may generate a novel splice site (10). In addition, variant rs8065832, was referenced in the Clinvar database (https://preview.ncbi.nlm.nih.gov/clinvar/) and Leiden Open Variation Database-LOVD (http://www.lovd.nl/3.0/home); In Clinvar, six reports were found for this variant, all of which categorize it as a benign mutation. However, LOVD yielded nine reports, which all categorized it as having an unknown effect.

**Figure 2:**
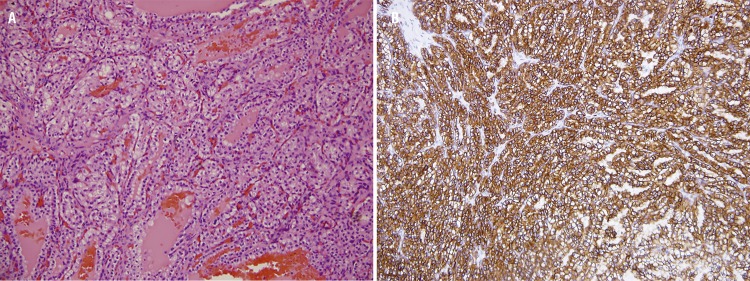
Light micrograph of a histologic specimen of human kidney. **a)** Hematoxylin and eosin of Clear cell carcinoma. **b)** Immunohistochemistry CK7 positive of Papillary-type renal carcinoma.

The follow-up abdominal MRI scans performed in April 2014 and November 2014 showed no significant changes compared to the previous scans. When the February 2015 MRI scan showed a tumor greater than 3cm, she underwent three right partial nephrectomies. In February 2015, the pathology report revealed that this tumor was multiple (#3) clear cell carcinoma, Fuhrman grade 2, one of them with positive margins (Figure-2a). In August 2015, she underwent two partial nephrectomies on the left kidney, which pathology reported as being two papillary-type renal carcinomas with negative margins (Figure-2b). The patient was classified as having right clear cell carcinoma (T1aN0M0) and left papillary-type renal carcinoma (T1aN0M0). In June 2017, the new MRI revealed a hypervascular solid lesion of 10mm in the posterior middle-third portion of the left kidney, which suggests the formation of a new tumor under actual surveillance.

## DISCUSSION

We reported the case of a patient without a familial background who presented with synchronous bilateral kidney tumors of different subtypes (ccRCC and pRCC). After genomic analyses of five genes that are relevant to renal cancer (VHL, MET, FLCN, SDHB, and SDHD), several polymorphisms were found, but most of them were not clinically relevant. The only exception was a variant in the FLCN gene, c.1062+6C>T (rs8065832). According to our in silico analyses, this mutation could affect the splicing process and function of the protein folliculin.

RCC is the most frequently diagnosed type of kidney cancer (2.4%) among all adult cancers and 90% of kidney tumors (11, 12). 75% of RCC cases are the clear cell subtype (ccRCC), followed by papillary subtype (pRCC), which is found in 15% of cases (13). Both tumor types originate in the proximal tubule. The patient reported in this study had one tumor of each subtype localized to each kidney (ccRCC in the right kidney and pRCC in the left one). According with Wiklund et al., (1) based on the Swedish Cancer Registry, the risk of having a bilateral synchronous renal tumor is 0.3% among the general population and 0.2% among women. However, this study did not report the risk related to the histologic subtype of renal tumor.

In our case, it is important to consider the presence of two different histological subtypes without a family history of RCC. In some patients, bilateral tumors could be considered metastasis. In this case, two different tumors were found (clear cell and papillary), which suggests that they arose independently. This symptom might also occur in a number of inherited forms of renal tumors such as von Hippel-Lindau and BHD syndrome (14). Additionally, it might occur in cases of non-familial bilateral and multifocal kidney disease, where the tumors arise independently, which might be the case for this patient.

Wiklund et al. (1), also reported the risk factors for bilateral metachronous tumors, which include being female and being under the age of 40 years old (RR 4.5, 95% CI 3.4 to 5.9 and RR 18, 95% CI 9.4 to 37.5 respectively). Although there was no report found for synchronous bilateral tumors like this case, but by extrapolating this information, we might consider that our patient fits both of these criteria.

Genome-wide association studies and next-generation sequencing methods have allowed for comprehensive molecular characterization of different cancers, including RCC. As previously mentioned, mutations in VHL, MET, FH, and FLCN genes have been associated with von Hippel-Lindau (VHL)-mediated ccRCC, hereditary type I pRCC, hereditary leiomyomatosis, type II pRCC and BHD. The most common form of sporadic ccRCC showed that VHL is affected by loss of heterozygosity at chromosome 3p in 90% of cases. However, somatic mutations or epigenetic silencing have been reported in >80% of these tumors, which is not surprising considering that this gene is a major driver of ccRCC pathogenesis (15, 16).

Pavlovich et al. (17) reported that among patients with BHD, 34% have chromophobic and 50% have oncocytic tumors. However, clear cell and papillary tumors were also present, thus suggesting a general carcinogenic risk for the kidney. In our patient, we have found an intronic variant (rs8065832) that potentially affects the splicing of the FLCN gene, which has been associated with BHD syndrome (11, 12). No studies have been performed to determine the relevance of this variant in regard to susceptibility to RCC. According to da Silva et al. (18) this variant was a low- penetrance susceptibility cancer allele in sporadic RCC and colorectal cancer (CRC). They also found this variant had a higher frequency among CRC cases compared to control patients (p=0.055), which suggests that this variant is in linkage disequilibrium with other pathogenic variants.

In this patient, the FLCN gene variant was not associated with other clinical symptoms usually related with FLCN mutation and BHD syndrome. These symptoms included benign skin tumors (fibrofolliculomas), pulmonary cysts, and/or recurrent pneumothorax. Bartram et al. have investigated the mutation in the tumor suppressor gene FLCN that is associated with renal cancer, but additional functional and biochemical validation are required to guide further research regarding the role of FCLN in RCC development (19).

Since we already performed a previous genomic study for this patient, we decided to crosscheck these results for breast, lung, and colorectal cancer and determine if there were any associations with RCC. We found that variant rs6983267 and the G/G genotype found in the patient is associated with higher risk of not only colorectal cancer, but an increased risk of thyroid, prostate, lung (20) and renal cancer (21).

We focused our attention on this case since this was a young patient with synchronous bilateral kidney tumor with two different histological subtypes. The patient was screened for germline mutations associated with renal cancer development. Our bioinformatics analyses identified a potentially relevant clinical variant. Since variant has a high frequency in some populations, it is possible that this variant might be a low-penetrance cancer susceptibility allele. The patient has the G/G genotype for rs6983267, which has been associated a higher risk for developing CRC and renal cancer.

To our knowledge, these polymorphisms (rs8065832 and rs6983267) have not been described previously in the context of ccRCC or pRCC. They might have pathogenic significance for a sporadic case of synchronous bilateral kidney tumors with different histological subtypes.

## CONCLUSION

Although these results are promising, future research is necessary to establish the role of FLCN variants rs8065832, and rs6983267 in regard to increasing susceptibility to renal cancer. It is important to remember that some genomic disturbances might be associated with synchronous bilateral kidney tumors with different histological subtypes, and it is important to perform a thorough genomic study. Furthermore, it is important that experts in urology, oncology, genomic, and other biomedical sciences work together as an interdisciplinary team to pursue future studies.

### Compliance with Ethical Standards

#### Ethical approval

We accomplished all international ethical standards. This is a case report and the information was taken only from the clinical records, additionally there was a computational analysis.

#### Informed consent

Informed consent was obtained from the participant.
